# Identifying common barriers and facilitators to linkage and retention in chronic disease care in western Kenya

**DOI:** 10.1186/s12889-016-3462-6

**Published:** 2016-08-08

**Authors:** Beth Rachlis, Violet Naanyu, Juddy Wachira, Becky Genberg, Beatrice Koech, Regina Kamene, Jackie Akinyi, Paula Braitstein

**Affiliations:** 1 Academic Model Providing Access to Healthcare (AMPATH), PO-Box 4606, Eldoret, 30100 Kenya; 2 University of Toronto, Dalla Lana School of Public Health, Toronto, ON Canada; 3 Ontario HIV Treatment Network, Toronto, ON Canada; 4 Department of Behavioral Sciences, Moi University, College of Health Sciences, School of Medicine, Eldoret, Kenya; 5 Department of Health Services, Policy and Practice, Program in Public Health, Brown University, Providence, RI USA; 6 Department of Medicine, Moi University, College of Health Sciences, School of Medicine, Eldoret, Kenya

**Keywords:** Barriers, Facilitators, Linkage, Retention, HIV, Tuberculosis, Hypertension, Kenya

## Abstract

**Background:**

Sub-Saharan Africa is increasingly being challenged in providing care and treatment for chronic diseases, both communicable and non-communicable. In order to address the challenges of linkage to and retention in chronic disease management, there is the need to understand the factors that can influence engagement in care. We conducted a qualitative study to identify barriers and facilitators to linkage and retention in chronic care for HIV, tuberculosis (TB) and Hypertension (HTN) as part of the Academic Model Providing Access to Healthcare (AMPATH) program in western Kenya.

**Methods:**

In-depth interviews and focus group discussions were conducted July 2012-August 2013. Study participants were purposively sampled from three AMPATH clinics and included patients within the AMPATH program receiving HIV, TB, and HTN care, as well as caregivers of children with HIV, community leaders, and healthcare providers. A set of interview guides were developed to explore perceived barriers and facilitators to chronic disease management, particularly related to linkage to and retention in HIV, TB and HTN care. Data were coded and various themes were identified. We organized the concepts and themes generated using the Andersen-Newman Framework of Health Services Utilization.

**Results:**

A total of 235 participants including 110 individuals living with HIV (*n* = 50), TB (*n* = 39), or HTN (*n* = 21); 24 caregivers; 10 community leaders; and 62 healthcare providers participated. Barriers and facilitators were categorized as predisposing characteristics, enabling resources and need factors. Many of the facilitators and barriers reported in this study were consistently reported across disease categories including personal drive, patient-provider relationships and the need for social and peer support.

**Conclusions:**

Our findings provide insight into the individual as well as broader structural factors that can deter or encourage linkage and retention that are relevant across communicable and non-communicable chronic diseases. The findings of the present study suggest that interventions should consider the logistical aspects of accessing care in addition to predisposing and need factors that may affect an individuals’ decision to seek out and remain in appropriate care.

## Background

Chronic diseases are on the rise in sub-Saharan Africa, in part because of improvements in life expectancy and better control of previously terminal illnesses such as HIV [1–4]. In Kenya, approximately 7.1 % of the population is living with HIV and HIV/TB co-infection is estimated to affect 48 % of new TB patients. However, NCDs are increasingly accounting for a higher proportion of national morbidity and mortality in Kenya [5]. Given the weak healthcare infrastructure and more traditional health-seeking behaviors of patients which have been primarily focused on treatment of acute disease, chronic diseases are presenting new and significant challenges to both patients and healthcare providers [4, 6, 7]. At the same time, people living with chronic infectious diseases including HIV and TB are already at risk for developing additional co-morbidities including non-communicable diseases [8].

When chronic diseases like HIV and hypertension (HTN) are left untreated, there is an increased risk of morbidity and mortality [9–12]. Furthermore, while TB is treatable, it can also be a chronic illness and scarring can be permanent [13]. In response to the rise of multiple co-morbidities, integrated care has become increasingly important [8, 14, 15]. While HIV, TB and HTN have different modes of transmission and associated risk factors [14], they also share certain characteristics including the need for consistent engagement in care. Given the need for regular follow-up, the establishment and maintenance of stable and consistent relationships between patients and the healthcare system is needed [14].

For most chronic illnesses, successful engagement in the continuum of care begins with testing and diagnosis, linkage into care followed by retention over the time. While initial linkage into care following testing is a crucial stage in the care continuum, many individuals are never successfully linked and thus may never receive the treatment, care and support they need. In our population-based setting, we found that while engagement in HIV care was high among those who were previously known to be HIV-positive, only 16 % of those newly diagnosed through home-based testing and counselling for HIV had linked to care during a median of 3 years [16]. Studies from other settings have also demonstrated poor linkage rates following testing [17–20]. In a recent study in South Africa, linkage rates for TB and hypertension were estimated at 52 and 50 % respectively suggesting that almost half of those screened do not link to care [21]. Not engaging in care following testing is a major missed opportunity for maximizing health outcomes, reducing morbidity and mortality, and minimizing ongoing transmission, in the case of communicable diseases such as HIV and TB [22, 23]. Additionally, once linked, continuous retention in chronic disease care is challenging for many. In the case of HIV, a systematic review demonstrated that retention in care at 36 months averages between 65–70 % [24] with attrition being mainly a result losses to follow-up. While there is a dearth of information available for retention in hypertension care programs, we do know that treatment success rates for TB remain poor with approximately 28 % of patients reported as lost to follow up [13].

To effectively address the challenges related to chronic disease management related to timely linkage and retention of patients, there is an urgent need to identify and understand the barriers and facilitators that pose challenges to engagement across various socio-cultural environments and disease categories. The latter is particularly important in the context of the increasing number of people living with HIV who are also living with co-morbid conditions [8, 25] and if the public health impact of earlier screening and diagnosis will be fully realized [13, 21]. We therefore conducted a qualitative study to explore the perspectives of patients, caregivers, and health care providers on engagement in chronic care for HIV, TB and HTN. Specifically, we explored common barriers and facilitators to linkage and retention in chronic disease care among patients currently enrolled in the Academic Model Providing Access to Healthcare (AMPATH) program in western Kenya.

## Methods

### Study Setting- The Academic Model Providing Access to Healthcare (AMPATH) program

**Fig. 1:**
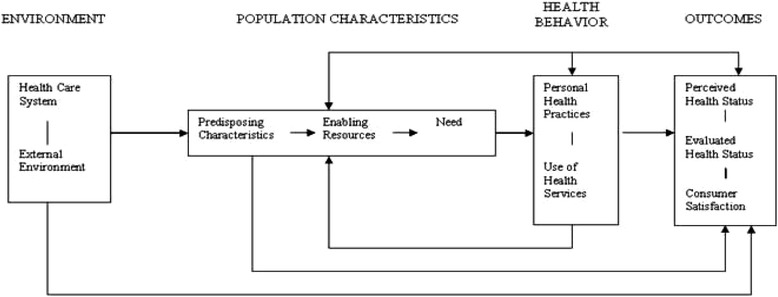
Map of Study Sites: This figure presents a map of all AMPATH sites in western Kenya and specifically highlights the three study sites: Chulaimbo, Teso and Turbo

The AMPATH program, headquartered in Eldoret, Kenya (about 350 km north-west of Nairobi) was initiated in 2001 as a joint partnership between Moi University School of Medicine, Moi Teaching and Referral Hospital (MTRH) [26, 27], and a consortium of North American universities led by Indiana University (IU) School of Medicine. The history, organizational structure, and health programs of AMPATH have been described elsewhere [28]. AMPATH provides technical support, mentorship and training to Kenyan medical faculty and staff with the aim of developing healthcare services in Kenya. AMPATH delivers care, provides education, and performs research in networks of urban and rural Ministry of Health hospitals, health centers, and dispensaries in western Kenya. AMPATH currently follows >85,000 HIV-positive patients in 22 sub-counties of 8 counties in western Kenya. All HIV and TB-related care and treatment are free at the point of service for patients. Patients are managed according to National Kenyan protocols, which are consistent with WHO guidelines. While AMPATH initially focused on patients infected with HIV, it has since expanded to provide maternal and child health services and chronic disease management, including diabetes and hypertension, to a catchment population of over 2 million persons [29, 30]. This study was undertaken in three AMPATH sites, namely Turbo, Teso, and Chulaimbo (Fig. 1).

### Target population

In order to gain additional perspectives on challenges to linkage and retention in care, we sought to include a broad range of participants including patients, community members and healthcare providers. More specifically, this study targeted patients within the AMPATH program including patients receiving HIV, TB, and hypertension care, as well as caregivers of children with HIV, community leaders (religious leaders, traditional healers, village elders, assistant chiefs), community health workers (CHWs), para-clinical staff (Nutritionist, Psychosocial, Outreach, Social work teams) and healthcare providers (nurses, clinical officers, medical officers).

### Study design

This was an exploratory qualitative study conducted between July 2012 and August 2013. Study participants were purposively sampled from three ethnically and geographically diverse AMPATH sites. In-depth interviews (*n* = 16) and focus group discussions (FGDs, *n* = 26) were used to collect data (See Table 1). The number of data collections was predetermined based on conventional guidelines that each sub-population of the study had a minimum of two sessions. Theoretical saturation was assumed based on the number of sessions completed per population. FGDs were held separately for each participant group and for men and women among patient groups with the exception of HTN and TB FGD which were mixed in Teso. A set of interview guides were developed to explore perceived barriers and facilitators to chronic disease management particularly for linkage and retention to HIV, TB and HTN care (See attached guides). Specific questions were asked about barriers and facilitators to linkage versus those related to retention and individually for each chronic disease of interest. In addition, basic socio-demographic information of age, gender, educational level and occupation was collected. Trained research assistants identified the target groups at AMPATH health facilities and informed them about the study. Health facility in-charges assisted with contacting the participants. Data collection was conducted by members of the research team at the Social Behavioral Team within AMPATH. While some respondents (e.g., AMPATH staff) knew of some members of the research team and understood that there was a need to inform the care program particularly related to chronic disease management, given the physical distance between AMPATH headquarters and the three rural sites, there was no prior relationship between participants and the researchers. The interview sessions and FGDs took approximately 1 h and were conducted in English, Swahili, Kalenjin, or Luo. All sessions were audio recorded and for the FGDs, scribes also took notes on session proceedings. At the end of each session participants were provided with transport reimbursement of 200 Kenyan Shillings (approximately $2.50 US). This research was program driven and was situated within the broader AMPATH Care Program with the goal of improving linkage and retention of patients within existing clinics. It was considered a low-risk rapid appraisal. Verbal consent was obtained prior to beginning data collection and again prior to commencing audio recording. While consent forms were not used, transcripts from the FGDs and in-depth interviews demonstrate agreement and consent to proceed with the data collection. For patients participating in FGD, they did not have to disclose the chronic disease status given that most participants knew each other’s conditions for they were recruited from specialty clinics that were caring for specific conditions. FGD were utilized only for patient groups as they were considered a more homogenous group. In-depth interviews were held with community leaders and provider groups only as they were considered a more heterogeneous group that was purposely selected based on their unique and comprehensive knowledge on the topics relevant for the present study. Finally, it is worth noting that his study was situated within a larger AMPATH Program protocol. Note that ethical approval for this study was obtained through an amendment of a larger AMPATH Program protocol that received ethical approval from the Institutional Research and Ethics Committee (IREC) of Moi University College of Health Sciences and Moi Teaching and Referral Hospital as well as the Indiana University Institutional Review Board (IRB).

**Table 1:** Participant Characteristics*

Site	PLWHA		HTN		TB		Caregiver	CHWS	Safety Nets	HCP
	Men	Women	Men	Women	Men	Women	Mixed	Mixed	Mixed	Mixed
Turbo	1	1	1	1	1	1	1	1	1	1
Teso	1	1	1 (mixed)		1 (mixed)		1	1	1	1
Chulaimbo	1	1	-		1		1	1	1	1
Site	Religious leader		Traditional healers		AMPATH/PHC in-charge		MOH in-charge		Village elder/assistant chief	
Turbo	1		1		1		1		1	
Teso	1		1		1		1		1	
Chulaimbo	1		2		1		1		1	

*PLWHA People Living With HIV/AIDS, *HTN* Hypertensive Patients, *TB* TB Patients, *Caregiver* for children living with HIV, *CHWS*Community Health Workers, *Safety Nets* includes nutritionists, outreach workers, social workers, psychosocial workers; HCP = Healthcare providers including clinical officers, nurses, pharmacists and lab technicians

### Data analysis

Recorded interviews and FGDs were transcribed and translated to English. The data were then coded and themes related to barriers and facilitators to HIV, TB and HTN linkage and retention were identified. Inductive and deductive data analysis approaches were used. Ideas from different interviews were pooled together and integrated into common themes. Concepts from these themes were generated and we used a conceptual model based on the Andersen-Newman Framework of Health Services Utilization to organize the presentation of the results. In the Andersen Newman Framework (Fig. 1), an individual’s access to and use of healthcare is a function of three main factors: 1) Predisposing Characteristics (socio-cultural characteristics of individuals that exist prior to their illness); 2) Enabling Resources (the logistical aspects of obtaining care, which can include personal, family and community resources); and 3) Need Factors (the most immediate cause of healthcare use from problems that generate the need for care) [31]. For validation, independent coding and identification of themes were conducted by five investigators. We started with a codebook that had a priori codes that were derived from the original question guide. The 5 investigators (VN, JW, RK, JO, BK), all women, worked independently to identify emerging inductive codes that were then added to the codebook as necessary although data was also interpreted based on pre-existing knowledge about the context, the study objectives and the identified themes. Training relating to qualitative data analysis including coding and thematic analysis was also provided. As well, all investigators involved in coding and interpretation had extensive experience in qualitative research methods. Of the investigators involved in coding and analysis, two have PhDs (1 in Sociology and 1 in Human Behavior) and three have Bachelor’s degree (2 in sociology and 1 in nutrition). The original codebook was created in unison (all 5 investigators were involved). The number of interviews were divided evenly among the 5 investigators. Each coder highlighted area of discrepancies and then met as a group to harmonize a response. Note that no software was used. The final write up consisted of summaries, interpretations and textual excerpts.

## Results

### Participant characteristics

A total of 235 participants including 110 individuals living with HIV (*n* = 50), TB (*n* = 39), or hypertension (*n* = 21); 24 caregivers; 10 community leaders; and 62 healthcare providers including community health workers (*n* = 28) participated in the study. There were no refusals to participate.

Based on the Anderson-Newman Framework of Health Services Utilization, barriers and facilitators were organized into three main categories: predisposing characteristics, enabling resources and need factors. Enabling resources (as barriers and facilitators) were most commonly reported. Most were common to both linkage and retention (described below) although there were some differences (e.g., Lack of Acceptance was reported as a barrier to linkage only; Media/peer influences a barrier to retention only) (Tables 2 and 3). Most barriers and facilitators were reported for HIV. See Tables 2 and 3 for list of barriers and facilitators for linkage and retention across disease categories.

**Table 2:** Barriers to Linkage and Retention to HIV, HTN and TB care

	Linkage			Retention		
	HIV	HTN	TB	HIV	HTN	TB
Predisposing Characteristics						
Lack of acceptance of status	X		X			
Forgetfulness	X	X		X	X	
Poor motivation	X	X	X	X	X	X
Lack of knowledge	X	X	X	X	X	X
Fear of taking drugs/expectations of treatment	X	X	X	X		X
Preference in alternative medicine	X		X	X	X	X
Older age/male gender	X			X		
Alcohol use/drug use	X			X	X	X
Patient attitudes to care/expectations of AMPATH				X		
Couple/family conflicts	X			X		
Promiscuity	X		X			
Cultural beliefs (e.g., association with witchcraft)		X		X		X
Disclosure				X		
Enabling Resources						
Perceived stigma	X	X	X	X		X
Relocation	X			X		X
Lack of finances/transport/food	X	X	X	X		X
Busy schedule/domestic chores	X			X		X
Ending support-food/lack of food support	X			X		
Distance/access to preferred health facility	X	X	X	X	X	X
Slow service/long queues	X	X	X	X	X	
Limited counselling	X					X
Patient-provider relationships	X		X	X		X
Poor information by providers				X	X	
Lack of case manager/availability of health providers	X				X	X
Stigma associated with health facility	X	X		X		
Cost of treatment	X	X			X	
Lack of specialized treatment		X				
Lack of medication/availability of over-the-counter medication		X			X	
Separate appointments for mother/child				X		
Drug packaging				X		
Competing interests from other NGOs				X		
Lack of partner support/discordance		X		X	X	X
Inadequate social support	X	X	X	X	X	X
Change in social status				X		
Media/peer influences				X	X	
Lack of care in institutions				X		
Need Factors						
Perceived health status	X	X		X	X	X
Worsening health status/severity of illness	X	X	X	X		X
Drug side effects	X			X	X	X
Long period of treatment/frequency of appointments/need for adherence			X	X		
Treatment regimens						X

**Table 3:** Facilitators to Linkage and Retention to HIV, HTN and TB care

	Linkage			Retention		
	HIV	HTN	TB	HIV	HTN	TB
Predisposing Characteristics						
Personal initiative	X	X	X	X	X	X
Education	X					
Disclosure	X			X		
Fear of losing a child/death/family responsibilities	X	X	X	X		X
Belief in treatment/improved health	X		X		X	
Enabling Resources						
Availability of education programs	X		X			
Community awareness/sensitization (e.g., through sports)	X	X	X			
Comprehensive counseling/appropriate referrals	X	X	X			
Availability of free drugs/services/subsidized treatment	X		X		X	
Integrated services (including food services, TB-HIV)	X		X	X		X
Availability and training of healthcare providers		X	X			
Accessibility of health facility	X	X	X		X	X
Good provider-patient relationship	X	X	X	X	X	X
Provider maintains confidentiality	X					
Government collaboration/health policy	X		X			
Follow-up care by CHWs for missed appointments	X			X		X
Transport facilitation to health facility/physical access	X					
Economic empowerment	X			X		
Peer-support testimonies/peer support	X	X	X	X	X	
Family/social support	X	X	X	X	X	X
Support from community leaders			X			
Need factors						
Severity of illness			X			
Fear of restarting treatment						X

### Barriers common to linkage and retention

#### Predisposing characteristics

Predisposing characteristics consisted of factors related to knowledge, attitudes and beliefs that hindered linkage and retention. While the majority of barriers were reported for linkage to and retention in HIV care, several barriers were commonly reported across disease categories and were common across linkage and retention.

Poor motivation was reported as a barrier to linkage and retention and was reported across all disease categories. Issues related to hopelessness and feeling tired were particularly challenging for retention. As one caregiver noted: “*Some people just get tired of taking their drugs and if there is no improvement in health, some may give up*” (Caregiver, Turbo). Another participant further elaborated: “…*let us take an example of these sexual workers, they are still having unprotected sex and all that so they get new strains of the virus…through that they take drugs but there is no change then they say, ‘I have been taking these drugs for more than a year now but there is no change, so what is the need for me to continue using the drugs” So they stop using them*” (HCP, Chulaimbo). Related to this is *forgetfulness* which was reported as a barrier to linkage and retention for HIV and HTN but not TB: “*We tend to forget that when we feel better after a while, we are cured…its human nature*” (Community Leader, Chulaimbo). “The other thing is laziness, wh*en someone has gone to bed and has not taken drugs for example, one will feel too lazy to get up and take the drugs (all laugh) or they forget to take them”* (HTN FGD participant, Turbo”).

A lack of knowledge and/or understanding around the need to engage in care also is a barrier to linkage and retention. “*…Someone may buy pain killers and feel much better and think they are OK. So if you tell such a person to go to the hospital, they’ll always procrastinate.*” (HTN FGD participant, Turbo)*.* An HIV positive patient noted that: “*Even when they come for treatment, they expect a quick recovery. But they don’t understand that this is a lifetime treatment. Because of this, they go back and don’t stop coming*” (PLWH FGD participant, Turbo). Again, this relates closely to lack of knowledge about the need to take medications: “*Somebody might be given a return date to go and monitor progress, when the condition improves he/she says, “Why should I go back to the doctor?”* (Religious Leader, Teso). Fear of taking drugs can also act as a major barrier to both linkage and retention. In terms of linkage to HIV, for example, one PLWH noted: “*there are some who fear coming for ‘andila’ (the name given to ARVs in the village) because they heard that once you start taking them, you can’t stop for the rest of your life…*” (PLWH FGD participant, Chulaimbo).

Many have preferences in alternative medicine. This was reported as a barrier to linkage for HIV and TB and all disease categories for retention. This may stem from fear of the medications themselves. *“Take medicines for a very long time but no sign of improvement, a person gives up or tries alternative medicine like herbal medicine”* (CHW FGD participant, Teso). Preferences for alternative forms of care may also be influenced by outside forces but can also be reflective of poor treatment literacy (e.g., understanding the need to adhere to appropriate medicines). For example, “…*advertisement may put across very appealing message that there is a doctor who has medicine that if you take for two weeks you be back to normal than these other conventional medicine*” (CHW FGD participant, Teso). The use of alternative forms of care may result in poor outcomes particularly when individuals fail to engage with appropriate care: “*There are people who are positive but have been brainwashed by herbal medicines. They are told herbs cure HIV so they dispose of their ARVs and use them. Some die due to this*” (Caregiver FGD participant, Turbo).

The use of alcohol/drugs was reported as a barrier. While misuse was reported for HIV linkage, it was a common barrier in terms of retention: “*They are told not to drink and take their medicine. They drink and forget or at times take their medicines while drunk*” (Community Leader, Chulaimbo). Another participant noted: “*One might stop taking their medication and resort to alcohol. …He does not have the time to come to the hospital or take his medication*” (TB FGD participant, Chuliambo).

Cultural beliefs and social norms that can negatively impact linkage and retention were also described: *“cultural issues such as a mother in-law and a son-in law being in the same queue”* (HCP, Teso)*.* Related to this are religious beliefs: *“These pastors tell people that you will be healed through prayers. A patient is told that you have been prayed for, so do not go back for those”* (HCP, Teso). Beliefs that their illnesses are a result of witchcraft were also described as a key barrier: “*…some symptoms are associated with witchcraft for example, a curse and they know that the symptoms can only be cured with certain systems …say church, if you are bewitched you probably need to see a sorcerer or something like that. While they are trying other interventions, it takes time for them to give up and come to the hospital…they dilly dally at home as they try those other avenues and self medicate, go to the church, go to the herbalist”* (HCP, Chulaimbo).

#### Enabling resources

Stigma was reported as a barrier for all categories with the exception of retention in HTN care. Fear of being recognized and discriminated against may deter an individual from initially linking to care as one HIV positive participant noted: “*I am not going, I will be seen*” (PLWH FGD participant, Chulaimbo). The same is true for retention, “…*They fear to be seen by their neighbors and will also disappear (all laugh).”* (HCP FGD participant, Turbo). While common for both linkage and retention to HIV care, stigma was also encountered in other chronic disease categories: “*You know if you are diabetic…you are discriminated and this causes you not to come to the hospital*” (Caregiver FGD participant, Chulaimbo).

A lack of finances was reported across all categories except as a barrier for retention for HTN care. Specific challenges were given related to the need for food “*There are some people for TB (pause) you know…when you are using those drugs you need to eat well. A person may not be having food…taking the drugs without the food weakens a person and that is why some people leave them*” (CHW FGD participant, Turbo). Broader issues with costs were also described “*The patient is supposed to come to the clinic on a monthly basis and it becomes expensive for many*” (HCP, Turbo). A religious leader pointed to the larger issue: *“…when somebody is poor it becomes a silent killer*…” (Religious Leader, Teso).

Busy schedules and domestic chores may act as a barrier, particularly in the context of widespread poverty. In particular, “*Other competing factors at home may affect appointments*” (CHW FGD participant, Teso). This can affect both linkage and retention particularly when confounded by financial challenges: “*She looks for food for the children. What can use for travel (to the clinic) instead she uses for food*” (CHW FGD participant, Turbo). Employment may also interfere with an individual’s ability to be retained: “*At times you might miss your appointment date due to working elsewhere. Most of us are casual laborers so you might go to a place where you are not close to a clinic”* (Caregiver FGD participant, Turbo).

Distance/access to the health facility was reported as a barrier for all diseases and as a barrier to both linkage and retention: *“…Distance and terrain of some places. If a person has no means (pauses) reaching here is a problem.*” (HCP, Chulaimbo). Distance to the clinic was particularly a challenge for individuals with financial constraints: *“You see, in the remote areas, transport to get to the health facility could be an issue. It could be Kenya shillings 100–300 on a motorbike. Most people will not afford this”* (HTN FGD participant, Turbo). A long distance and poor access can negatively affect the health of patient. The negative impacts of distance and poor access were also described: “*For TB (Pauses) people who come from far and they are suffering from TB, the distance covered is long and the patient will be tired*” (CHW FGD participant, Teso). Another explained that the *“…mama who has to walk for 6 or 7 km is too unfair*” (Religious Leader, Teso).

The distance to the clinic may be particularly a problem particularly when participants also have to deal with slow service and long queues: “…*One can wait for the doctor from 6 am to 6 pm and does not see the doctor after all that long wait*” (HTN FGD participant, Turbo). As one Caregiver noted, “*If they come and realize that the services are slow, they might get upset and go away, never to return”* (Caregiver FGD participant, Turbo). Furthermore, the cost of treatment was reported as a barrier for linkage and retention for HIV. The cost of the drugs themselves was a barrier: “*Drugs are expensive*” (HCP, Chulaimbo). Another participant noted that “*Some just lack money. There is no need to come to the clinic because they have no money to buy drugs”* (CHW FGD participant, Turbo).

Patient-provider relationships were a commonly reported factor affecting linkage and retention for both communicable and non-communicable chronic diseases. The nature of the relationship was described as being particularly important “*It also depends on health providers. The way they talk to us. You may get one who is so good and talks to you so well but at time you may get another one that is arrogant, harass you. You give up”* (HTN FGD participant, Teso). Many participants spoke to issues with maintaining confidentiality: *“…Health providers may know this client very well and we are human beings. This provider may go home and expose the status (*i.e.*, HIV)*” (HCP, Teso). When patients miss a visit or are late for a visit, they fear to be scolded by their health providers: *“…There are those who start but stop along the way. In most cases the health care providers contribute. Maybe the way they talk to you is bad. There was a time I defaulted. I missed an appointment date for the child so when I came the doctor was too harsh on me…I went and stayed for some years…. So the manner in which the doctor talks to the patient makes a lot”* (Caregiver FGD participant, Turbo). If a patient misses a visit or is late for an appointment, the clinic staff may scold them: “*When you come late, you are told to go back and add another year before you come. The health care providers chase you away”* (Caregiver FGD participant, Turbo). Another individual noted that: “*health providers are very strict on adherence and if you missed to take medicines as required they are harsh on you and some people fear that. If they have done such mistakes they prefer staying at home*” (PLWH FGD participant, Teso).

Stigma associated with the facility itself was reported as a barrier to linkage and retention with “*AMPATH being particularly associated with HIV*” (PLWH FGD participant, Teso). This may cause individuals to not link: “*Some just stay at home without seeking medical attention. Some have even passed on because they fear coming to AMPATH*” (PLWH FGD participant, Teso). Participants described their experiences and coping strategies: “…*in our clinic the CDM patients, hypertensive and diabetic, they queue with the HIV patients…when the patients are queuing, they want to tell their neighbour, the next person, you know, I am not HIV+, I have a CDM card (lots of laughs). And the next time, they won’t come to the clinic because they are queuing with the HIV positives* (HTN FGD participant, Turbo). Stigma associated with AMPATH was not unique to patients as one healthcare provider eluded: “*Another problem is the AMPATH thing. The community knows that anyone who is found on the premises is HIV+. …even the staff*” (HCP, Turbo).

#### Need factors

The perceived severity of disease and symptoms experienced may also influence whether an individual engages in care. For example, an individual may not understand or recognize disease symptoms: “… *generally within the Teso community they have not identified the symptoms of TB or even know that TB is a killer… Most people who have TB take it as a normal cough; others see it as a chest problem… They deny TB as a name.”* (Religious Leader, Teso). “*It’s only when people are critically ill to the point that they cannot walk on their own that they can decide to come to the health facility* [Laughter]” (HTN FGD participant, Turbo)*.* This also speaks to an individuals’ perceived health status: “*Maybe one came when he was very weak and after using medication he improves and thinks he is cured*” (TB FGD participant, Chulaimbo). Feeling better was a particular challenge for retention: “*when one improves he thinks he is healed and stops coming to the clinic. He feels he is of good health*” (Traditional Healer, Chulaimbo).

This has implications for treatment effectiveness and poor health outcomes: *“....We have seen some patients that when they take medicines their health improves and they believe that they are now completely healed. They leave medicines and for some time their health deteriorates and they come back to the clinic whereby sometimes they even die*” (CHW FGD participant Teso).

Similar reports have been given for TB: “*There are some drugs like ones for TB. When you start using them you feel dizzy and you might even collapse. Some patients fear when they experience the side effects, then they stop using drugs*” (Caregiver FGD participant, Turbo). Related to retention is the issue of having to take drugs long-term: “*There are some who fear coming for ARVS drugs because they heard that once you start taking them, you can’t stop for the rest of your life. They wonder and say, ‘Will I take these drugs till I die? This is stress and I can’t’ [Laughter]. So there are people who fear taking drugs”* (PLWH FGD participant, Chulaimbo).

Lack of partner support/discordance was reported as a barrier to linkage to TB care and was reported across disease categories as a barrier to retention: *“A lack of support from partners matters. My husband said to me, ‘You die with your pressure so that I re-marry. What are you still doing? Just die’ [Laughs]. It is not until I looked for my own money that I came to the hospital”* (CHW FGD participant, Teso). Fear of being blamed, in the case of HIV, also was brought up: “*It is an issue among youth and couples…especially when one partner is tested, when she goes back home, she doesn’t know whether the partner is positive or negative so she decides to keep quiet because if she says anything, it will be concluded that maybe she is the one who brought it.”* (HCP, Teso). One caregiver noted that without proper support from one’s partner, retention in care is a challenge: “*You may not be able to meet all your needs or the husband might not be supportive. You then decide to stop treatment and sit back*” (Caregiver FGD participant, Teso).

Inadequate social support, more generally was a commonly reported barrier. In some cases, this can affect whether an individual is able to get to the clinic: e.g. “*some have been identified but they are too weak but can’t get somebody to accompany them to the hospital”* (HCP, Chulaimbo). Inadequate support may also lead an individual to not get better particularly if there is no one to help take care of them or remind them to take their drugs: “*there are some children who are HIV positive and quite a good number of them are orphans so it is a caregiver who takes care of them….and to some extent the condition of the child is worse and if the caregiver doesn’t have a heart to take care of that child, he/she becomes fatigued and stops bringing the child for care”* (HCP, Chulaimbo). Furthermore, challenges within households can also present challenges: “*In some families there is a lot of violence that sometimes discourages a woman to continue with drugs”* (Religious Leader, Chulaimbo).

### Facilitators

In general, there were fewer facilitators compared to barriers and most facilitators were reported for linkage to HIV care.

#### Predisposing characteristics

While poor motivation was a barrier to linkage and retention, having a strong personal initiative was reported as a facilitator to linkage and retention for all disease categories. In terms of linkage, taking responsibility for one’s health can drive individuals to seek out care as one participant noted: *“I think it is personal responsibility. People are concerned about their health and they have realized that chronic diseases like hypertension and diabetes are not diseases of the rich - Anybody can get it. So they are coming to the clinic because they are concerned.”* (HCP, Turbo)*.* One patient further noted that “*people like us who want our health to be prolonged, come here*…” (PLWH FGD participant, Teso). Personal initiative was also described as a facilitator to takings medications as prescribed: “*In my opinion, it is very important for me to take drugs because if I don’t, I might die. It is better to take good care of my health by completing the dose as instructed by the doctor*” (TB FGD participant, Chulaimbo).

A belief in treatment, and in AMPATH, was also an important facilitator. In terms of linkage, an individual may come for care “*because they are assured that his health being taken care of. Then AMPATH has been on the ground for long and people are aware of the services offered.”* An improvement in health also works to facilitate retention. As one traditional healer noted: *“Once people start using the medication, they improve. The fact that TB is treatable motivates people to come for treatment”* (Traditional Healer, Chulaimbo).

Family responsibilities and fear of losing a child were common facilitators to linkage for all disease categories and for retention to HIV and TB care. *“Another reason is that we have children at home that depend on us. You do not wait to get wasted until you are not able to provide for your family”* (HTN FGD participant, Teso)*.* It was also important to protect children from getting sick: “*so when you come here you are told if you take your child to clinic you can save your child from getting HIV…Like me I fear to lose a child like I did, he was my first born* (Caregiver FGD participant, Teso).

#### Enabling resources

These were the most common type of facilitators for linkage and retention to care. Good provider-patient relationships were the most commonly reported facilitator to care: *“There was a time when patients were picked from their homes to the hospital. When one fails to come to the clinic, he is followed up. The healthcare providers love the patients and this motivates them to come for care”* (Traditional Healer, Chulaimbo*).* Being treated with respect was important for patients: “*Staff are warm. They help with counseling from testing through to treatment*” (PLHW FGD participant, Teso). Related to this is how patients are spoken to: “*the nurse who gives us medication talks to us nicely. I don’t know whether I am the only one she talks to well but she treats me so well. She asks me several questions concerning my health before she gives me the drugs*” (TB FGD participant, Chulaimbo).

Accessibility of the health facility was also reported as a facilitator. Bringing services to the community was noted: “*AMPATH brought very good services to Chulaimbo*” (Religious Leader, Chulaimbo). Proximity to one’s home is also an important facilitator: “*Personally, I am very happy coming here because it’s a clinic next to our homes, expenses have been reduced. I commend AMPATH for that. It has made it possible for us to attend the clinic on a regular basis*” (HTN FGD participant, Turbo). Another noted: *“I enrolled here because it was near*” (TB FGD participant, Teso).

The provision of integrated services was cited as a facilitator to care for HIV and TB linkage and retention. Including food services was particularly important: “*We also have these patients who will feel like for sure they have been helped for the six months they participated in the food program and they feel appreciated and they will come back to the clinic, because they say that they were helped at the time of need”* (HCP, Turbo). This was particularly important context of widespread poverty: “*Free food especially at this time of famine. There is no food at home and you are so weak that you cannot provide for yourself. You don’t care even if people see you at AMPATH it is about the disease. It is not your choice. These people are very good. If you call, them the driver just brings you food or they make follow ups to see how you are doing*” (CHW FGD participant, Teso). The integration of TB-HIV care was also seen as positive: “*OK, for TB patients, since the integration of the TB/HIV clinic… the number of TB patients coming to the clinic is steadily rising and also the retention of these clients is perfect and the adherence to medication is good. So I think I can relate this to the kind of services we are offering, that it is centered to one person who is always available to attend to the client”* (HCP, Turbo).

Related to this are the services available: “*Some clients told me that they go to other health facilities where weight is only checked and given ARVs. AMPATH takes all the vitals, tests blood pressure, pulse, weight and temperature (pauses)…everything. It gives very good drugs”* (PLWH FGD participant, Chulaimbo). The provision of free/subsidized services was also described as a facilitator: “*It’s a place where they can get drugs at the revolving pharmacy; the prices are lower compared to the prices they pay with in chemists in town”* (HCP, Turbo).

Peer support/testimonies and family and social support were reported facilitators for the majority of categories with the latter being an important facilitator for linkage and retention in all disease categories. The role of peer testimonies and improvements in health could work to influence individuals to link with care: “*I had a friend that was wasted and people never thought that he would recover but I met him afterwards. He was so healthy and was able to do his work. He gave me counselling and that is how I got to come to the hospital”* (HTN FGD participant, Teso). It could also work to encourage one’s own acceptance of their illness and it many ways they “*themselves become the facilitators*” (Traditional healer, Chulaimbo). Another participant noted that “*It helps… Because it encourages, you talk about some issues (pause). You encourage each other*” (CHW FGD participant, Turbo). Peer testimonies can help individuals to disclose their own status: *“....I joined a support group where I got to know how to disclose after counselling. If you want to live long with HIV (pauses) you talk about. Don’t keep it for yourself”, I was told”* (PLWH FGD participant, Chulaimbo).

Family and social support can help individuals to come to terms with their illness and link to care: “*If there is no improvement, his own people will advise him to go to the hospital”* (Traditional Healer, Chulaimbo). Social support also works to encourage positive behaviors that are important for maintaining one’s health: *“Yes, you must be given time to think and make a personal decision. I was tested and given one week to go and think over it. My mother really encouraged me”* (PLWH FGD participant, Chulaimbo)*.* As some participants noted, it can depend on the family: *“If the family understands then you will not suffer a lot of trauma*” (PLWH FGD participant, Teso). Importantly, children and families could also remind patients to take their medications: “*When I’m held up with some domestic chores my children remind me that have you taken drugs? “I have heard or I’m going to take them now I do reply”’ There is support”* (HTN FGD participant, Teso).

#### Need factors

Severity of illness and fear of restarting treatment were two need factors considered as facilitators to linkage and retention to TB care, respectively.

## Discussion

Enabling resources as barriers were the most commonly reported factors affecting linkage and retention. In addition to adding to the literature, our findings provide additional insight on common barriers and facilitators that can deter or encourage linkage and retention across multiple chronic diseases. Importantly, the number of comorbid conditions is rising in LMICs when an increasing number of individuals managing more than one chronic condition that requires continuous engagement in care [8, 25]. The findings of the present study highlight facilitators and barriers that are common across chronic diseases and can inform interventions that consider the logistical factors that can facilitate or inhibit engagement in care.

Many predisposing factors could be either a barrier (e.g., poor motivation) or a facilitator (e.g., personal initiative) depending on the situation and/or context. Attitudes including self-efficacy and beliefs in treatment effectiveness have been shown to be important for adherence to care and treatment and successful self-management of one’s illness [32–34]. Attitudes towards engagement in care can be influenced by other factors such as knowledge/or lack of knowledge about the disease and treatment options as well as the need for adherence [34–37]. In addition, while believing in treatment effectiveness can lead to positive health seeking behaviours [38], an improved health and the belief that treatment is no longer necessary can be a barrier to care.

Patient-provider relationships are an enabling resource that can be both a barrier and facilitator, depending on the nature of the interactions. The importance of these relationships and its role in retention in care [33, 39], has been well studied in high income settings [40–42] and in recent years, its importance, in LMICs, has emerged. Consistent with other studies [33, 43–45], our findings identified specific aspects of patient-provider relationships that can impact on engagement in care including issues with maintaining confidentiality. The use of rough or exposing language by healthcare workers has been shown to be an important reason for disengaging from HIV care [46, 47]. The fear of being yelled at if missing a visit has been shown to be a strong predictor for feeling reluctant to return to the clinic [47]. A recent study from our setting suggests that perceived physician communication behaviours are associated with adherence to HIV care [48]. Physician trust has been previously been associated with adherence to diabetes management [49, 50] and a trusting patient-provider relationship has also been associated with improved HIV testing for patients with TB [51] and improved chronic disease management more generally [52]. Sensitization training for healthcare workers should include strategies to improve communication and interpersonal skills to build patient trust and encourage long term engagement in care.

Interestingly, while individual predisposing characteristics, enabling resources and need factors were reported as barriers or facilitators, it is likely combinations of these factors work together in a complex sequence of events [47]. For example, in the case of HIV, disclosure of one’s status has been recognized as a double-edged sword [53], having the potential to yield needed social support but can also result in stigmatization/abandonment [53–56]. In the present study, this extended to beliefs about stigma associated with the health facility. The labelling of AMPATH as an HIV organization can dissuade individuals from seeking care for other conditions like hypertension. Importantly for patients who chose to go to care elsewhere due to a fear of being recognized at a clinic closer to home, this may mean that they have to travel further to receive care. This can result in higher transport costs [46, 57–60]. However, while retention can be improved when care is closer located closer to patient homes [57, 61–64], individuals continue to face numerous challenges and complexities when negotiating care; these need to be addressed and considered in intervention/strategic planning.

The integration of care and treatment services across an array of diseases may be a vital strategy to encourage engagement in appropriate care especially given that similar self-management skills are needed across chronic diseases [15]. Making a range of services available in one place, including food support and education programs, can help to simplify care and treatment [14] and facilitate engagement for those who may have otherwise not linked as was demonstrated in this study. While there the goals of integrative programs may vary with disease-specific needs, broadly they seek to support self-management skills, medication adherence, coping skills and the promotion of self-efficacy [15]. AMPATH’s Bridging Income Generation with Provision of Incentives for Care (BIGPIC) uses an integrated approach to address barriers specific to socioeconomic status for individuals living with diabetes and hypertension and works to improve positive health seeking behaviours. While useful, integrated approaches require continuous evaluation to explore their acceptability and identify potential negative impacts (e.g., not wanting to be identified as HIV positive if queuing in the same line). Furthermore, programs should be targeted and tailored for specific contexts and settings [15] and support individuals with logistical and financial challenges [56].

Social support was a facilitating enabling resource for linkage and retention to care. Family influences [34, 65] have been shown to be important predictors of adherence to care and treatment [34, 36, 66–68]. The desire to be alive and be able to support their families and see their children grow up is a strong motivator for patients [69, 70]. Family members are an important source of support [65, 71, 72] and given that a large proportion of illness care takes place in the home [73], remains one of the most important supporting mechanisms for individuals living with chronic diseases [73–75]. There is a need to further elucidate the type and nature of familial relationships that work to encourage engagement in care. At the same time, the health and well-being of family members and caregivers supporting individuals with chronic diseases can also be affected [72, 73, 75–79], suggesting that strategies that address the burden on caregivers (e.g., psychosocial support) are also needed.

Participants also identified peer support as a facilitator to care. In addition to developing a supportive connection with individuals with similar lived experiences, peer support can improve adherence to treatment [67, 78], promote self-management [80], coping skills and problem solving [14], reduce experiences of stigma and isolation [81], improve beliefs in treatment, self-efficacy [82] and feelings of hope [67, 72]. Peer support groups are common across many diseases and have been shown to have positive impacts on clinical and social outcomes. In diabetes management for example, peer support has resulted in decreases blood pressure while improving contact with the healthcare team and overall self-management [83, 84]. The use of peer navigators in the HIV literature has shown to improve access to care including retention in care as well as improving acceptance of one’s status and disclosure [85, 86]. Importantly, peers may be more approachable than healthcare providers. In Malawi for example, patients living with HIV noted that in addition to being reliable sources of support and essentially acting as role models, expert patients are better able to understand them and respect confidentiality compared to other types of health workers [87].

There are a few limitations worth noting. This was a qualitative study and we acknowledge that our findings cannot be generalized to the wider Kenyan population. It mainly presented views of communities studied and was limited to perceptions about AMPATH health facilities. There may be other barriers and facilitators that were not captured but that are relevant for linkage. Furthermore, in the present study only patients who had linked and engaged in care were included. Importantly, individuals who did not link to care may be different and have different perspectives on the key barriers and facilitators to linkage and retention.

## Conclusions

We qualitatively explored barriers and facilitators to engagement in HIV, TB and HTN care in western Kenya. Numerous factors were consistent across disease categories including the importance of personal drive and patient-provider relationships. Future research should work to identify other barriers and facilitators that may be common to linkage and retention across diseases as well as evidence-based strategies to support timely engagement in care while considering logistical and financial barriers. Integrated service delivery for chronic diseases, may offer once such strategy given the increasing prevalence of NCDs among the general population and among those living with HIV although such models should be rigorously evaluated.

## Abbreviations

AMPATH, Academic Model Providing Access to Healthcare; ART, antiretroviral therapy; BIGPIC, bridging income generation with provision of incentives for care; CHW, Community Health Worker; FGD, focus group discussion; HCP, Healthcare provider; HIV, human immunodeficiency virus; HTN, hypertension; IREC, Institutional Research and Ethics Committee; IU, Indiana University; LMIC, low and middle income countries; MTRH, Moi teaching and referral hospital; NCD, non communicable disease; PLWH, person living with HIV; TB, tuberculosis; USAID, United States Agency for International Development; WHO, World Health Organization

## References

[1] Venkat Narayan KM, Miotti PG, Anand NP, Kline LM, Harmston C, Gulakowski R, Vermun SH (2014). HIV and noncommunicable disease comorbidities in the era of antiretroviral therapy: a vital agenda for research in low- and middle-income country settings. J Acquir Immune Defic Syndr.

[2] Geneau R, Hallen G (2012). Toward a systematic research agenda for addressing the joint epidemics of HIV/AIDS and noncommunicable diseases. AIDS.

[3] Levitt NS, Steyn K, Dave J, Bradshaw D (2011). Chronic noncommunicable diseases and HIV-AIDS on a collision course: relevance for health care delivery, particularly in low-resource settings-insights from South Africa. Am J Clin Nutr.

[4] de Graft AA, Unwin N, Agyemang C, Allotey P, Campbell C, Arhinful D (2010). Tackling Africa’s chronic disease burden: from the local to the global. Global Health.

[5] Kenyan National Bureau of Statistics. Kenya Integrated Household Budget Survey (KIHBS) 2005/2006. Nairobi: http://statistics.knbs.or.ke/nada/index.php/catalog/8.

[6] Boutayeb A (2006). The double burden of communicable and non-communicable diseases in developing countries. Trans R Soc Trop Med Hyg.

[7] Remais JV, Zeng G, Li G, Tian L, Engelgau MM (2013). Convergence of non-communicable and infectious diseases in low- and middle-income countries. Int J Epidemiol.

[8] Marais BJ, Lonnroth K, Lawn SD, Migliori GB, Mwaba P, Glaziou P, Bates M, Colagiuri R, Zijenah L, Swaminathan S, Memish ZA, Pletschette M, Hoelscher M, Abubakar I, Hasan R, Zafar A, Pantaleo G, Craig G, Kim P, Maeurer M, Schito M, Zumla A (2013). Tuberculosis comorbidity with communicable and non-communicable diseases: integrating health services and control efforts. Lancet Infect Dis.

[9] Pastakia SD, Ali SM, Kamano JH, Akwanolo CO, Ndege SK, Buckwalter VL, Vedanthan R, Bloomfield GS (2013). Screening for diabetes and hypertension in a rural low income setting in western Kenya utilizing home-based and community-based strategies. Global Health.

[10] Steyn K, Sliwa K, Hawken S, Commerford P, Onen C, Damasceno A, Ounpuu S, Yusuf S, INTERHEART Investigators in Africa (2005). Risk factors associated with myocardial infarction in Africa: the INTERHEART Africa study. Circulation.

[11] Bangsberg DR, Charlebois ED, Grant RM, Holodniy M, Deeks SG, Perry S, Conroy KN, Clark R, Guzman D, Zolopa A, Moss A (2003). High levels of adherence do not prevent accumulation of HIV drug resistance mutations. AIDS.

[12] Chalker J, Andualem T, Minzi A, Ntaganira J, Ojoo A, Waako P, Ross-Degnan D (2008). Monitoring adherence and defaulting for antiretroviral therapy in 5 east African countries: an urgent need for standards. J Int Assoc Physicians in AIDS Care.

[13] World Health Organization. Global tuberculosis report 2013

[14] Van Olmen J, Schellevis F, Van Damme W, Kegels G, Rasschaert F (2012). Management of chronic diseases in sub-Saharan Africa: cross-fertilization between HIV/AIDS and Diabetes Care. J Trop Med.

[15] Swendeman D, Ingram BL, Rotheram-Borus (2009). Common elements in self-management of HIV and other chronic illnesses: an integrative framework. AIDS Care.

[16] Genberg B, Naanyu V, Wachira J, Hogan JW, Sang E, Nyambura M, Odawa M, Duefiled C, Ndege S, Hogan J, Braitstein P: Linkage to and engagement in HIV care in western Kenya: An observational study using population-based estimates from home-based counseling and testing. Lancet HIV, 2014 (in press). 10.1016/S2352-3018(14)00034-4 PMC4302338 25621303

[17] Rosen S, Fox MP (2011). Retention in HIV care between testing and treatment in sub-Saharan Africa: a systematic review. PLoS Med.

[18] Clouse K, Page-Shipp L, Dansey H, Moatlhodi B, Scott L, Bassett J, Stevens W, Sanne I, Van Rie A (2012). Implementation of Xpert MTB/RIF for routine point-of-care diagnosis of tuberculosis at the primary care level. S Afr Med J.

[19] Kayigamba FR, Bakker MI, Fikse H, Mugisha V, Asiimwe A, van der Loeff SMF (2012). Patient enrolment into HIV care and treatment within 90 days of HIV diagnosis in eight Rwandan health facilities: a review of facility-based registers. PLoS One.

[20] Kranzer K, Govindasamy D, Ford N, Johnston V, Lawn SD (2012). Quantifying and addressing losses along the continuum of care for people living with HIV infection in sub-Saharan Africa: a systematic review. J Int AIDS Soc.

[21] Govindasamy D, Kranzer K, van Schaik N, Noubary F, Wood R, Walensky RP, Freedberg KA, Bassett IV, Bekker LG (2013). Linkage to HIV, TB and Non-communicable disease care from a mobile testing unit in cape town, South Africa. PLoS One.

[22] Daniel OJ, Oladapo OT, Alausa OK (2006). Default from tuberculosis treatment programme in Sagamu, Nigeria. Niger J Med.

[23] YV D l, Evans D, Page-Shipp L, Barnard A, Sanne I, Menezes CN, Van Rie A (2013). Linkage to care and treatment for TB and HIV among people newly diagnosed with TB or HIV associated TB at a large, inner city South African hospital. PLoS One.

[24] Fox MP, Rosen S (2015). Retention of adult patients on antiretroviral therapy in low- and middle-income countries: systematic review and meta-analysis 2008–2013. J Acquir Immune Defic Syndr.

[25] Nguyen KA, Peer N, Mills EJ, Kengne AP (2015). Burden, determinants, and pharmacological management of hypertension in HIV-positive patients and populations: a systematic narrative review. AIDS Rev.

[26] Einterz RM, Kimaiyo S, Mengech HN, Khwa-Otsyula BO, Esamai F, Quigley F, Mamlin JJ (2007). Responding to the HIV pandemic: the power of an academic medical partnership. Acad Med.

[27] Mamlin JJ, Kimaiyo S, Nyandiko W, Tierney W. Academic institutions linking access to treatment and prevention, in Perspectives and Practice in Antiretroviral Treatment. W.H. Organization, Editor, 2004, World Health Organization Geneva. [http://www.who.int/hiv/pub/prev_care/en/ampath.pdf]

[28] Kimaiyo S, Were MC, Shen C, Ndege S, Braitstein P, Sidle J, Mamlin J (2010). Home-based HIV counseling and testing in western Kenya. East Afr Med J.

[29] Bloomfield GS, Kimaiyo S, Carter EJ, Binanay C, Corey GR, Einterz RM, Tierney WM, Velazquez EJ (2011). Chronic noncommunicable cardiovascular and pulmonary disease in sub-Saharan Africa: An academic model for countering the epidemic. Am Heart J.

[30] Bloomfield GS, Hogan JW, Keter A, Sang E, Carter JE, Velazquez EJ, Kimaiyo S (2011). Hypertension and obesity as cardiovascular risk factors among HIV seropositive patients in western Kenya. PLoS One.

[31] Andersen R, Newman JF (1973). Societal and individual determinants of medical care utilization in the United States. Milbank Mem Fund Q Health Soc.

[32] Bernal H, Woolley S, Schensul JJ, Dickinson JK (2000). Correlates of self efficacy in Diabetes self-care among Hispanic adults with diabetes. Diabetes Educ.

[33] Johnson MO, Chesney MA, Goldstein RB, Remien RH, Catz S, Gore-Felton C, Charlebois E, Morin SF (2006). Positive provider interactions, adherence self-efficacy, and adherence to antiretroviral medications among HIV-infected adults: a mediation model. AIDS Patient Care STDS.

[34] Rachlis B, Mills EJ, Cole DC (2011). Livelihood security and adherence to antiretroviral therapy in low and middle income settings: a systematic review. PLoS One.

[35] Al Sayah F, Majumdar SR, Williams B, Roberston S, Johnson JA (2013). Health literacy and health outcomes in diabetes: a systematic review. J Gen Intern Med.

[36] Mills EJ, Nachega JB, Bangsberg DR, Singh S, Rachlis B, Wu P, Wilson K, Buchan I, Gill CJ, Cooper C (2006). Adherence to HAART: a systematic review of developed and developing nation patient-reported barriers and facilitators. PLoS Med.

[37] Zhang NJ, Terry A, McHorney CA (2014). Impact of health literacy on medication adherence: a systematic review and meta-analysis. Ann Pharmacother.

[38] Siril H, Fawzi MC, Todd J, Wyatt M, Kilewo J, Ware N, Kaaya S. Hopefulness fosters affective and cognitive constructs for actions to cope and enhance quality of life among people living with HIV in Dar Es Salaam, Tanzania. Journal of the International Association of Providers in AIDS Care 2014 (in press). 10.1177/2325957414539195 PMC7903482 24963087

[39] Scoenthaler A, Montaque E, Baier Manwell L, Brown R, Schwartz MD, Linzer M. Patient-physician racial/ethnic concordance and blood pressure control: the role of trust and medication adherence. Ethnicity and Health 2013, in press. 10.1080/13557858.2013.857764 PMC4031314 24266617

[40] Miller D, Steele Gray C, Kuluski K, Cott C. Patient-centred care and patient reported measures: lets look before we leap. Patient 2014 (in press). 10.1007/s40271-014-0095-7 PMC4529474 25354873

[41] Brennan N, Barnes R, Calnan M, Corrigan O, Dieppe P, Entwistle V (2013). Trust in the health-care provider-patient relationship: a systematic mapping review of the evidence base. International J Qual Health Care.

[42] Lo B (1999). The patient-provider relationship: opportunities as well as problems. J Gen Intern Med.

[43] Gourlay A, Birdthistle I, Mburu G, Iorpenda K, Wringe A (2013). Barriers and facilitating factors to the uptake of antiretroviral drugs for prevention of mother-to-child transmission of HIV in sub-Saharan Africa: a systematic review. J Int AIDS Soc.

[44] MacPherson P, MacPherson EE, Mwale D, Squire SB, Makombe SD, Corbett EL, Lalloo DG, Desmond N (2012). Barriers and facilitators to linkage to ART in primary care: a qualitative study of patients and providers in Blantyre, Malawi. J Int AIDS Soc.

[45] Wachira J, Middlestadt SE, Vreeman R, Braitstein P (2012). Factors underlying taking a child to HIV care: implications for reducing loss to follow-up among HIV-infected and -exposed children. SAHARA J.

[46] Rachlis B, Ahmad F, van Lettow M, Muula AS, Semba M, Cole DCC (2013). Using concept mapping to explore why patients become lost to follow up from an antiretroviral therapy in the Zomba District of Malawi. BMC Health Serv Res.

[47] Ware NC, Wyatt MA, Geng EH, Kaaya SF, Agbaji OO, Muyindike WR, Chalamilla G, Agaba PA (2013). Towards an understanding of disengagement from HIV treatment and care in sub-Saharan Africa: a qualitative study. PLoS Med.

[48] Wachira J, Middlestadt S, Reece M, Peng CY, Braitstein P (2014). Physician communication behaviours from the perspective of adult HIV patients in Kenya. International J Qual Health Care.

[49] Bell RA, Arcury TA, Ip E, Grzywacz JG, Nguyen H, Kirk JK, Saldana S, Quandt SA (2013). Correlates of physician trust among older rural adults with diabetes. Am J Health Behav.

[50] Mancuso JM (2010). Impact of health literacy and patient trust on glycemic control in an urban USA population. Nurs Health Sci.

[51] Corneli A, Jarrett NM, Sabue M, Duvall S, Bahati E, Behets F, Van Rie A (2008). Patient and provider persepectives on implementation models of HIV counselling and testing for patients with TB. Int J Tuberc Lung Dis.

[52] Murray B, McCrone S. An integrative review of promoting trust in the patient-provider relationship. Journal of Advanced Nursing 2014, (in press). 10.1111/jan.12502 25113235

[53] Birbeck GL, Chomba E, Kvalsund M, Bradbury R, Mang’ombe C, Malama K, Kaile T, Byers PA, Organek N, RAAZ Study Team (2009). Antiretroviral adherence in rural Zambia: the first year of treatment availability. Am J Trop Med Hyg.

[54] Norman A, Chopra M, Kadiyala S (2007). Factors related to HIV disclosure in 2 South African communities. Am J Public Health.

[55] Kalichman SC, Ramachandran B, Catz S (1999). Adherence to combination antiretroviral therapies in HIV patients of Low health literacy. J Gen Intern Med.

[56] Merten S, Kenter E, McKenzie O, Musheke M, Ntalasha H, Martin A (2010). Patient-reported barriers and drivers of adherence to antiretrovirals in sub-Saharan Africa: a meta-ethnography. Trop Med Int Health.

[57] McGuire M, Munyenembe T, Szumilin E, Heinzelmann A, Le Paih M, Bouithy N, Pujades-Rodriquez M (2010). Vital status of pre-ART and ART patients defaulting from care in rural Malawi. Trop Med Int Health.

[58] Kebede A, Wabe NT (2012). Medication adherence and its determinants among patients on concomitant tuberculosis and antiretroviral therapy in south west Ethiopia. N Am J Med Sci.

[59] Harries AD, Zachariah R, Lawn SD, Rosen S (2010). Strategies to improve patient retention on antiretroviral therapy in sub-Saharan Africa. Trop Med Int Health.

[60] Labhardt ND, Balo JR, Ndam M, Manga E, Stoll B (2011). Improved retention rates with low-cost interventions in hypertension and diabetes management in a rural African environment of nurse-led care: a cluster-randomised trial. Trop Med Int Health.

[61] Geng EH, Nash D, Kambugu A, Zhang Y, Braitstein P, Christopoulous KA, Muyindike W, Bwana MB, Yiannoutsos CT, Petersen ML, Martin JN (2010). Retention in care among HIV-infected patients in resource-limited settings: emerging insights and new directions. Curr HIV/AIDS Rep.

[62] Chan AK, Mateyu G, Jahn A, Schouten E, Arora P, Mlotha W, Kambanji M, van Lettow M (2010). Outcome assessment of decentralization of antiretroviral therapy provision in a rural district of Malawi using an integrated primary care model. Trop Med Int Health.

[63] Mukora R, Charalambous S, Dahab M, Hamilton R, Karstaedt A (2011). A study of patient attitudes towards decentralization of HV Care in an urban clinic in South Africa. BMC Health Serv Res.

[64] O’Connor C, Oshi R, Jaffer A (2011). Loss to follow-up of stable antiretroviral therapy patients in a decentralized down referral model of care in Johannesburg, South Africa. J Acquir Immune Defic Syndr.

[65] Ware NC, Idoko J, Kaaya S, Biraro IA, Wyatt MA, Agbaji O, Chalamilla G, Bangsberg DR (2009). Explaining adherence success in sub-Saharan Africa: an ethnographic study. PLoS Med.

[66] Makoae LN, Portillo CJ, Uys LR, Dlamini PS, Greeff M, Chirwa M, Kohi TW, Naidoo J, Mullan J, Wantland D, Durrheim K, Holzemer WL (2009). The impact of taking or not taking ARVs on HIV stigma as reported by persons living with HIV infection in five African countries. AIDS Care.

[67] Masquillier C, Wouters E, Mortelmans D, le Roux BF (2014). Families as catalysts for peer adhernece support in enhancing hope for people living with HIV/AIDS in South Africa. J Int AIDS Soc.

[68] Paz-Soldan VA, Alban RE, Jones CD, Oberhelman RA (2013). The provision of and need for social support among adult and pediatric patients with tuberculosis in Lima, Peru: a qualitative study. BMC Health Serv Res.

[69] Remien RH, Hirky AE, Johnson MO, Weinhardt LS, Whittier D, Le GM (2003). Adherence to medication treatment: a qualitative study of facilitators and barriers among a diverse sample of HIV+ men and women in four US cities. AIDS Behav.

[70] Watt MH, Maman S, Earp JA, Eng E, Setel PW, Golin CE, Jacobson M (2009). “It’s all the time in my mind”: Facilitators of adherence to antiretroviral therapy in a Tanzanian setting. Soc Sci Med.

[71] Klotz LK (2010). Hope in relation to nursing interventions for HIV-infected patients and their significant others. J Assoc Nurses AIDS Care.

[72] Harris GE, Larsen D (2008). Understanding hope in the face of an HIV diagnosis and high-risk behaviours. J Health Psychol.

[73] World Health Organization. Community home-based care in resource-limited settings: a framework for action. 2002. http://www.who.int/hiv/pub/prev_care/isbn9241562137.pdf

[74] Shaibu S (2013). Experiences of grandmothers caring for orphan grandchildren in Botswana. J Nurs Scholarsh.

[75] Seeley J (1993). The extended family and support for people with AIDS in a rural population in south west Uganda; a safety Net with holes?. AIDS Care.

[76] Akintola O, Hangulu L (2014). Infection control in home-based care for people living with HIV/AIDS/TB in south Africa; an exploratory study. Glob Public Health.

[77] Awadalla AW, Ohaeri JU, Al-Awadi SA, Tawfiq AM (2006). Diabetes mellitus patients’ family caregivers’ subjective quality of life. J Natl Med Assoc.

[78] Belayachi J, Himmich S, Madani N, Abidi K, Dendane T, Zeggwagh AA, Abougal R (2014). Psychological burden in inpatient relatives: the forgotten side of medical management. QJM.

[79] Chepngeno-Langat G (2014). Entry and re-entry into informal care-giving over a 3-year prospective study among older people in Nairobi slums, Kenya. Health Soc Care Community.

[80] Pearson CR, Micek MA, Simoni JM, Hoff PD, Matediana E, Martin DP, Gloyd SS (2007). Randomized control trial of peer-delivered, modified directly observed therapy for HAART in Mozambique. J Acquir Immune Defic Syndr.

[81] Horter S, Stringer B, Venis S, du Cros P (2014). “I can also serve as an inspiration”: a qualitative study of the TB&me blogging experience and its role in MDR-TB Treatment. PLoS One.

[82] Kaponda CP, Jere DL, Chimango JL, Chimwaza AF, Crittenden KS, Kachingwe SI, McCreary LL, Norr JL, Norr KF (2009). Impacts of a peer-group intervention on HIV-related knowledge, attitudes and personal behaviours for urban hospital workers in Malawi. J Assoc Nurses AIDS Care.

[83] Fisher EB, Boothroyd RI, Coufal MM, Baumann LC, Mbanya JC, Rotherman-Borus MJ, Sanguanprasit B, Tanasugarn C (2012). Peer support for self-management of diabetes improved outcomes in international settings. Health Aff.

[84] Baumann LC, Nakwagala F, Nambuya A (2010). Peer support for adults with diabetes in rural Uganda: champions and partners. Int J Behav Med.

[85] Bradford JB, Coleman S, Cunningham W (2007). HIV system navigation: an emerging model to improve HIV care access. AIDS Patient Care STDS.

[86] Living Society of British Columbia. Peer Navigator Program: Interim Evaluation Report: April 1 2011-May 31, 2012

[87] Tenthani L, Cataldo F, Chan AK, Bedell R, Martiniuk AL, van Lettow M (2012). Involving expert patients in antiretroviral treatment provision in a tertiary referral hospital HIV clinic in Malawi. BMC Health Serv Res.

