# Correction to: Identifying common barriers and facilitators to linkage and retention in chronic disease care in western Kenya

**DOI:** 10.1186/s12889-018-5904-9

**Published:** 2018-08-10

**Authors:** Beth Rachlis, Violet Naanyu, Juddy Wachira, Becky Genberg, Beatrice Koech, Regina Kamene, Jackie Akinyi, Paula Braitstein

**Affiliations:** 1 Academic Model Providing Access to Healthcare (AMPATH), PO-Box 4606, Eldoret, 30100 Kenya; 2 Dalla Lana School of Public Health, University of Toronto, Toronto, ON Canada; 3 Ontario HIV Treatment Network, Toronto, ON Canada; 4 Department of Behavioral Sciences, Moi University, College of Health Sciences, School of Medicine, Eldoret, Kenya; 5 Department of Health Services, Policy and Practice, Program in Public Health, Brown University, Providence, RI USA; 6 Department of Medicine, Moi University, College of Health Sciences, School of Medicine, Eldoret, Kenya

## Correction

After the publication of the original article [1], it was highlighted that Fig. 1 was incorrectly labeled. Figure 1 should refer to the Andersen-Newman Framework for Health Services Utilization. Additionally, the reference to Fig. 1 following the brief description of the three AMPATH study sites within the Study Setting- The Academic Model Providing Access to Healthcare (AMPATH) program section under the Methods should be removed. This Correction articles shows the correct Fig. 1.

**Fig. 1:**
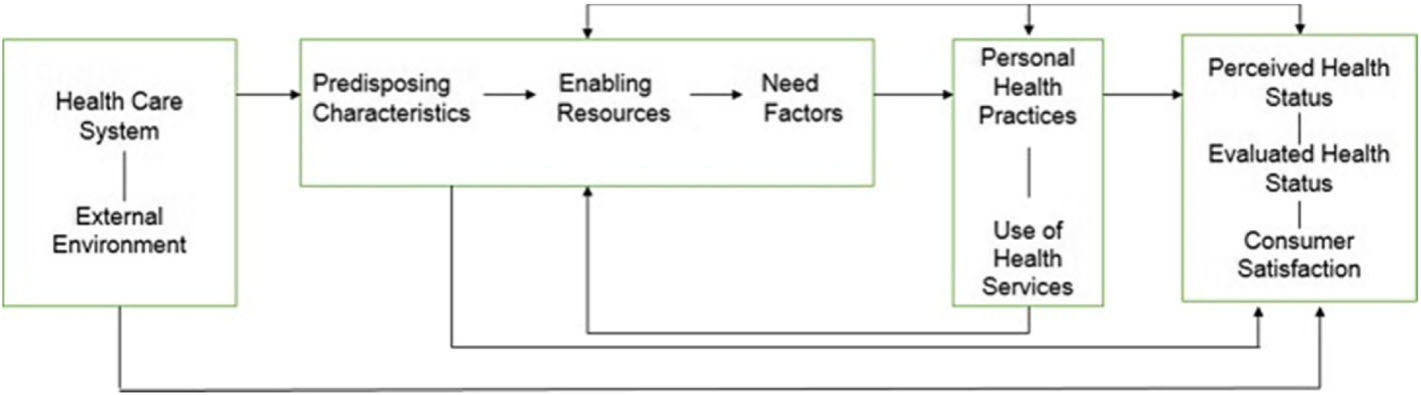
Andersen-Newman Framework for Health Services Utilization
